# The Role of Relational Embeddedness in Enhancing Absorptive Capacity and Relational Performance of Internationalized SMEs: Evidence From Mainland China

**DOI:** 10.3389/fpsyg.2022.896521

**Published:** 2022-05-24

**Authors:** Liwen Zhou, Zhong Chen, Michael Yao-Ping Peng

**Affiliations:** ^1^Research Center of Open Economics and Trade, Fuzhou University of International Studies and Trade, Fuzhou, China; ^2^School of Business, Xiamen Institute of Technology, Xiamen, China; ^3^Business School, Foshan University, Foshan, China

**Keywords:** absorptive capacity, organizational learning, relational embeddedness, relational memory, relational performance, social capital

## Abstract

The social network in the organizational learning process is a critical knowledge source to realize superior performance. The purpose of this study is to examine the relationship between relational memory, relational embeddedness, and absorptive capacity, and their impact on the relational performance of small and middle enterprises (SMEs). This study empirically verifies the research framework from 223 Chinese internationalized SMEs. The results confirm previous studies that indicate positive correlations among relational embeddedness, relational memory, and absorptive capacity. The results also indicate that relational embeddedness and relational memory have positive effects on relational performance. The findings show that relational memory plays a mediating role in the relationship between relational embeddedness, absorptive capacity, and relational performance.

## Introduction

Recently, many scholars have paid attention to firm performance, especially in strategic management. There are also many insights on firm performance (Pinho and Prange, 2016; Rui et al., 2017). As discussed in prior studies, the firm performance reveals that firms deal with operation with effect by adapting their resources and capabilities (Zhou and Li, 2010; Pinho and Prange, 2016). Due to increasing changes in the competition environment, firms cannot merely respond to firm performance, also have to focus on the relational performance with partners, especially for small and middle enterprises (SMEs, enterprises with a number of employees <200 can be classified as SMEs) (Cegarra-Navarro, 2007; Liu et al., 2018). As argued by some scholars, improved core competence/capabilities is/are the prerequisite for better firm performance (Saranga et al., 2018). However, there still have a lack of understanding of the development of relational performance (Cheung et al., 2010), while relational performance can be referred to as an indicator to measure relationship changes among organizations and reflect differences in the sources of enterprise knowledge (Chen et al., 2013; Albort-Morant et al., 2018). This study thus investigates key antecedents of SMEs' relational performance from perspectives of organizational capability and organizational learning.

This study summarizes the existing organizational learning literature on performance by analyzing the effect, of one critical factor that can enhance relational performance: knowledge absorptive capacity (AC) (Camisón and Forés, 2010), which can be defined as the ability of organizations to handle external knowledge and integrating internal knowledge and to control external business opportunities and create new values. Many studies have indicated that the absorptive capacity contributes to organizational innovation (Cohen and Levinthal, 1990; Tsai, 2001; Fang et al., 2011; Albort-Morant et al., 2018), organizational performance improvement, inter-organizational learning enhancement (Cheung et al., 2010), as well as facilitating knowledge transfer (Liu, 2012). However, few scholars have discussed how firms construct absorptive capacity, that is, what processes or mechanisms should be built and what knowledge or resources should be accumulated to effectively develop absorptive capacity (Lane et al., 2006; Albort-Morant et al., 2018). Scholars have illustrated that the development of enterprise competence lies in the characteristic of path dependence (Andersson et al., 2007; Alinaghian et al., 2020), and it's a dynamic continuous set of knowledge and information integration, of which the development process will be affected by internal and external factors to change the nature and appearance of competence (Pinho and Prange, 2016; Albort-Morant et al., 2018), and further respond to and adapt to vicissitudes in the external environment (Wu et al., 2020). A similar view has been proposed in most studies, indicating that both predisposing factors within and outside the organization should be focused on when considering the development of absorptive capacity in order to improve the study framework (Lane et al., 2006; Liu, 2012; Rakthin et al., 2016; Flor et al., 2018; Alinaghian et al., 2020). Hence, the study aims to explore the impact of absorptive capacity on relational performance from internal and external perspectives.

With regard to the external perspective, some scholars argued that firms are able to enhance their AC through knowledge acquisition via social networks in the literature on social capital (Evangelista and Hau, 2009; Aribi and Dupouët, 2015; Albort-Morant et al., 2018; García-Villaverde et al., 2018; Liu et al., 2018). Although scholars have verified the impact of the relational dimension of social capital on knowledge acquisition and knowledge learning and applied it in the interdisciplinary field (Fang et al., 2011; Chen et al., 2013; Liu et al., 2018), few studies have contributed to our understanding of the relational dimension of social capital in the procedure of knowledge processing (Pinho and Prange, 2016; Alinaghian et al., 2020). In particular, most of them deals with relational dimension such as relational embeddedness (Dezi et al., 2019), which promote the importance of understanding how SMEs access significant knowledge source to achieve knowledge and information advantage (Andersson et al., 2007; Evangelista and Hau, 2009; Ferraris et al., 2018; Alinaghian et al., 2020; Wu et al., 2020). Based on above-mentioned statements, this study uses relational embeddedness as a significant variable for the measurement of relational dimension (Dhanaraj et al., 2004; Ferraris et al., 2018), and defines it as the relational intensity between organizations and other players in the social network. Specifically, Riikkinen et al. (2017) indicated that less is known whether enterprises in the international context differ from the domestic context in terms of the process of resource-capability-competence (Flor et al., 2018; Dezi et al., 2019; Alinaghian et al., 2020). They found that there was a significant difference between domestic and international contexts. According to this perspective, the relational performance of SMEs lies on the strength of relational embeddedness to access more knowledge, which means that SMEs should use this knowledge to foster their AC, to improve relational performance. Thus, this study aims to explore the impact of relational embeddedness on AC and relational performance.

According to organizational learning theory, Cohen and Levinthal (1990) argued that the accumulation of organizational memory (prior knowledge) is an important driving factor for firms to be familiar with (Lane et al., 2006; Cegarra-Navarro, 2007; Liu et al., 2018; Kmieciak, 2019), identify and acquire new external information and knowledge (Albort-Morant et al., 2018; Ferraris et al., 2018; Flor et al., 2018; Riikkinen et al., 2018). In other words, the organizational memory accumulated by the firm can affect the construction of absorptive capacity (Camisón and Villar-López, 2011; Wu et al., 2020). However, few studies in the past have explored what kind of knowledge or organizational memory needs to be accumulated so as to help firms with absorptive capacity construction (Liu, 2012; Kmieciak, 2019). According to the achievements studied by scholars, inter-organizational memory—also called relational memory—is accumulated through formal and informal interaction between the manufacturers with external partners (Andersson et al., 2007; Albort-Morant et al., 2018), embedded in a particular situation among the organizations (Cheung et al., 2010; Fang et al., 2011), and stored in the carrier within a few organizations (such as documents and computers) (Cegarra-Navarro, 2007; Camisón and Villar-López, 2011), employed by internal personnel in the organization, with an organization as the unit of analysis, also known as relationship knowledge (Selnes and Sallis, 2003; Johnson et al., 2004; Liu, 2012; Albort-Morant et al., 2018). Therefore, in order to understand how firms acquire external knowledge, store such knowledge and transform it into AC, this study aims to investigate the role of relational memory between absorptive capacity and relational performance.

## Literature Review and Hypotheses

### Absorptive Capacity

It lacks in figuring out how to do innovative knowledge seek from the external environment, and do replacement of the knowledge creation activities of firms, and how to make combination strengthening of existed knowledge base and external knowledge (Lane et al., 2006; Camisón and Forés, 2010; Riikkinen et al., 2018), and make enhancement of innovation activities and performance by conversion, all of which are correlated with the AC (Cohen and Levinthal, 1990; Rakthin et al., 2016; Wu et al., 2020). Lane et al. (2006) defined AC as the capability of firms effectively use external knowledge in three successive processes, including (1) identifying and understanding useful, external knowledge by means of exploratory learning; (2) acquiring new useful knowledge by means of transformative learning; (3) applying the acquired knowledge to novel knowledge and creation of commercial consequence by means of exploitative learning (Camisón and Forés, 2010; Liu, 2012; Albort-Morant et al., 2018; Peng and Lin, 2021).

Escribano et al. (2009) claimed that AC depends on the current knowledge assets of firms, which is emphasized new knowledge recognization, digestion, and utilization. Thus, most of the knowledge assets are reflected in products, procedures, and personnel (Camisón and Forés, 2010; Peng and Lin, 2021). In regard to conceptualization, most scholars illustrated that AC is a latent construct of multidimensions (Zahra and George, 2002; Flattena et al., 2011; Kang and Lee, 2016; Flor et al., 2018; Limaj and Bernroider, 2019). For the first-order level, AC contains a series of four capabilities, which include acquisition, assimilation, transformation, and exploitation (Albort-Morant et al., 2018). The capabilities of acquisition and assimilation form the potential absorptive capacity (PAC). The capabilities of transformation and exploitation make up the realized absorptive capacity (RAC) (Zahra and George, 2002; Albort-Morant et al., 2018; Limaj and Bernroider, 2019; Wu et al., 2020). Typically, PAC mainly makes acquisition and digestion of knowledge obtained externally, and successively creates new knowledge by means of internal processes, while RAC converts internal knowledge, which is utilized in response to environmental changes (Zahra and George, 2002; Flattena et al., 2011; Limaj and Bernroider, 2019). Namely, the components of PAC and RAC can be conceptualized as constructs of second-order level (Camisón and Forés, 2010).

PAC serves as the capacity for the manufacturer to acquire and internalize knowledge (Zahra and George, 2002), that is, the manufacturer identifies and acquires external knowledge which is conducive to organizational operation, thus improving the capacity to analyze, process, interpret and make comprehension of information obtained outside (Flor et al., 2018). Ritala and Hurmelinna-Laukkanen (2013) demonstrated that PAC can effectively improve the performance of organizational innovation, and if the manufacturer can share knowledge obtained with partners, it can contribute to knowledge base construction with existing and emerging technologies and concepts (Flor et al., 2018). Tu et al. (2006) believed that knowledge acquisition is an integral part of PAC, which allows the manufacturer to identify and capture external knowledge to make expertise augmentation in specific areas for the organization (Albort-Morant et al., 2018). However, in the case of insufficient PAC, it is unavailable for SMEs to collaborate with partners in designing, developing, and processing innovation (Flor et al., 2018), which would inhibit cooperation synergy between SMEs and their partners and further lead to relationship performance reduction. Moreover, with a higher level of PAC, SMEs are able to know the development trends of new products and the needs of new customers in a better way (Albort-Morant et al., 2018), enabling partner manufactures to jointly invest in the process of knowledge creation so as to produce products that are superior to their competitors (Chen et al., 2013). Therefore, this study proposes the following:

*H1: PAC positively correlates with relational performance*.

RAC is the capacity for the manufacturer to transfer and apply external knowledge (Zahra and George, 2002), that is, the manufacturer combines new knowledge with existing knowledge, and based on this, it refines, extends, and expands available capacity, making it conducive to applying knowledge that has been combined and transferred to business operation. The new knowledge effectiveness of the manufacturer depends on the knowledge base accumulated and learned by the organization in the past (Albort-Morant et al., 2018). New ideas can be more effectively infused through RAC in order to increase the capacity for the organization of figuring out new ideas, enhancing creativity, and identifying new opportunities (Cepeda-Carrion et al., 2012). The transfer capacity in RAC can effectively transfer knowledge accumulated within and among the organizations, and enrich the stock of the knowledge base for the manufacture, facilitating the development of new products and meeting new market demands. In addition, the capacity for knowledge application is a key process in RAC. Knowledge acquired from internal and external sources can promote the development of innovation capacity for the manufacturer (Cepeda-Carrion et al., 2012; Albort-Morant et al., 2018; Wu et al., 2020). Zahra and George (2002) stated that the innovation of products and processes by manufacturers can affect financial performance. The transfer capacity in RAC can help the manufacturer develop new cognitive models and change existing organizational processes so that new processes can be implemented effectively (Albort-Morant et al., 2018). In addition, organizations with good application capacity can enable the manufacturer to integrate new knowledge into the organization's new products, technologies, services, and operation management in a more effective way. Based on this, a hypothesis is deduced as follows in this study:

*H2: RAC positively correlates with relational performance*.

### Relationship Memory

Relationship memory is a special form of organizational memory (Johnson et al., 2004; Cegarra-Navarro, 2007; Liu, 2012). When an organization interacts, learns, and cooperates with external partner manufacturers, it integrates and accumulates the information it shares and acquires into shared relationship-specific memory (Selnes and Sallis, 2003; Albort-Morant et al., 2018), and stores it in the organizational hierarchy of individual manufactures, which can be shared and searched by employees within the organization (Li, 2006; Cegarra-Navarro, 2007; Liu et al., 2018). Fang et al. (2011) believed that the difference between relationship memory and organizational memory lies in: (1) The process of organizing memory: Relationship memory is the relevant knowledge of collective insight, belief, conduct routine, procedure, policy, etc. accumulated via interaction among organizations, which is shared by partner organizations (Johnson et al., 2004; Cegarra-Navarro, 2007). (2) Connotation of information and knowledge: Relationship-specific memory is the shared knowledge accumulated by organizations and external partners via social interaction (Selnes and Sallis, 2003; Cegarra-Navarro, 2007), in which both parties share and interpret knowledge and information owned by each other that can help both parties internalize each other'as implicit knowledge and skills (Kmieciak, 2019).

Under the condition of limited resources, inter-organizational learning is a vital channel for manufacturers to acquire significant knowledge and capacity (Fang et al., 2011; Albort-Morant et al., 2018; Liu et al., 2018). Inkpen and Currall (2004) argued that inter-organizational learning can be roughly classified into two categories, namely “learning from a partner” and “learning about a partner.” Based on the two kinds of organizational learning (Liu, 2012; Riikkinen et al., 2018), three knowledge bases of cumulative learning can be distinguished, including the concepts of functional, environmental, and interactional knowledge stores (Inkpen and Currall, 2004; Johnson et al., 2004). Interactional knowledge store (IKS) refers to the knowledge issues related to partner interactions that reflect the psychosocial components of inter-organizational relationships such as communication, coordination, conflict management, and the development and application of cooperative plans. Functional knowledge store (FKS) refers to the knowledge issues related to functional management of the supply chain, including the scope of cooperation with suppliers, such as cost deduction, quality control, operation and production, logistics, inventory management, and product development. Environmental knowledge store (EKS) refers to knowledge that is relevant to the external operating environment, including factors of the overall task environment, such as competitive behavior, market situations, and vicissitudes in legal norms (Johnson et al., 2004; Liu, 2012).

IKS contributes to the establishment of trust and commitment in organizational relationships (Johnson et al., 2004), while trust and commitment are the basis for maintaining inter-organizational relationships and relationship learning (Selnes and Sallis, 2003), which facilitates the exchange and merger of knowledge among organizations (Nahapiet and Ghoshal, 1998; Tsai and Ghoshal, 1998; Liu et al., 2018), and prompts individual manufactures to acquire, internalize, apply and merge the knowledge of their partners, thus contributing to the construction of AC for individual manufactures. Functional knowledge arising from the relationship management of value chain creates core process of competitive advantages for the organization (Camisón and Villar-López, 2011; Filieri and Willison, 2016), while inter-organizational learning is an important way to expand knowledge and resource base (Fang et al., 2011), which helps the manufacture with AC construction (Volberda et al., 2010). Jiang et al. (2010) illustrated that the diversity of the alliance portfolio determines the types of functional knowledge that can be obtained by the manufacturer (Lavie and Rosenkopf, 2006). By accumulating marketing knowledge or functional knowledge such as research and development, the manufacturer can expand the market area, increase value creation, and apply it to the construction of future core competence (Jiang et al., 2010). After the knowledge integrated through joint learning activities is absorbed and interpreted by the manufacturer, it can generate new insights which contribute to the reconfiguration of the existing knowledge base (Raisch et al., 2009). Besides, the abundant FKS facilitates the more effective application and extension of knowledge by the manufacturer (Johnson et al., 2004; Lavie and Rosenkopf, 2006).

EKS is the knowledge relevant to an external operating environment that is acquired and accumulated in the process of cooperating and learning with external partners, including market knowledge such as competition and customers (Flor et al., 2018). Lane et al. (2006) argued that the market environment an organization is in determines whether the manufacturer will continue to invest in the construction of AC, that is, the knowledge of the market environment is fundamental to the construction of AC. De Luca and Atuahene-Gima (2007) illustrated that the more extensive the market knowledge base (Cegarra-Navarro, 2007), the more effective merger the manufacturer can make of different knowledge elements so as to improve the identification of market opportunities and to gain a wider and more perceptive view (Chen et al., 2013). The deeper the market knowledge base is, the more complex and delicate the accumulated knowledge of customers and competitors is, the more effective the manufacturer can be in responding to customer problems and demands (Liu et al., 2018), as well as figuring out the advantages and reactions of potential competitors (Camisón and Villar-López, 2011; Filieri and Willison, 2016). These contribute to the emergence of new ideas. Based on the above arguments, a hypothesis is deduced as follows in this study:

*H3: Relational memory positively correlates with (a) PAC and (b) RAC*.

The intention of knowledge sharing and the dissemination and accumulation of knowledge depends greatly on the relationship among involved organizations (Chen et al., 2013; Kmieciak, 2019). That's why a mutually trusted and well-connected partnership among all stakeholders is considered one of the most important strategic assets. In other words, firms must develop their relational capabilities through sustained knowledge creation and aggregation (Fang et al., 2011). Due to knowledge mobility, many firms acquire and accumulate knowledge resources through establishing partnerships in face of increasingly fierce competition (Chen et al., 2013; Kmieciak, 2019). The popularity of relationship learning has caused comparable concerns for the issue of relationship memory because of its capability of storing the relationship learning results and propelling the knowledge retrieval and exchange (Camisón and Villar-López, 2011; Kmieciak, 2019). That is, through information sharing, dissemination, and interpretation among partners, relationship learning represents the process of creating knowledge (Cegarra-Navarro, 2007). In this process, relationship memory consists of interactional, functional, and environmental knowledge acquired through the establishment of partnerships. Johnson et al. (2004) argued that relational memory derives from the process of relational learning. Information is contained in a series of interactions between buyers and sellers, such as preferences, personality, personal characteristics, special appellation, terminology, little tricks, habits, etc., from the representative of the other side, and it goes through the process of sharing, understanding and spreading by each other (Fang et al., 2011), thus making the related departments in the individual organization be familiar with the interaction with partner organizations (Cegarra-Navarro, 2007), as well as producing interactional knowledge storage (Filieri and Willison, 2016). In addition, activities come from the two sides in the functional aspects, such as cost reduction, quality control, production and operation activities, logistics, inventory management, and product development (Camisón and Villar-López, 2011), and have been operating and formed a piece of routine in the context of imperceptible relational learning. And these activities and conventions will be available for the improvement of value created by the partnership. Furthermore, as for knowledge from the external business environment faced by both partners, such as market conditions, competitive behaviors, and regulatory changes (Kmieciak, 2019), once changes occur in the business environment, it needs to form a consensus on the characteristics of the current environment via information and cognition share with each other by partner organizations, thus reducing the impact of environmental uncertainty.

*H4: Relational memory positively correlates with relational performance*.

### Relational Embeddedness (RE)

Relational embeddedness denotes the oftenness of communication and interaction among network members. Higher relation embeddedness will lead to a larger amount of knowledge (Evangelista and Hau, 2009; Ferraris et al., 2018; Alinaghian et al., 2020), and more solutions or novel ideas can also be developed to achieve the collective goal (Evangelista and Hau, 2009). From the angle of the social network, prior studies of knowledge transfer consider the strength of the network as the core of the network formed by a series of social relations (Moran, 2005; Aribi and Dupouët, 2015; García-Villaverde et al., 2018). Specifically, the relational dimension of social capital (i.e., relational embeddedness) (Dezi et al., 2019) refers to the overall pattern resulting from ties and interaction among network members (Moran, 2005; Gilsing and Duysters, 2008; Alinaghian et al., 2020). It plays a critical role in accessing resources and information, including a tight network system generated via information exchange, the efficiency and promptness of information acquisition, the referral of interests, and the formulation of groups norms (Gilsing and Duysters, 2008; Ferraris et al., 2018; Liu et al., 2018). To accomplish the organizational goals, members in a network structure need to exchange and produce knowledge, involving knowledge search, retrieval, sharing, absorption, and transformation (Dhanaraj et al., 2004; Nielsen, 2005; Park and Glaister, 2009; Alinaghian et al., 2020). Dhanaraj et al. (2004) contended that organizations can exchange knowledge and information through market, hierarchy, or a hybrid. The international joint venture is a typical example of such a hybrid, which expounds on the relationships among organizations with three factors in RE: trust, the strength of connection, and a shared system (Nielsen, 2005; Park and Glaister, 2009; Liu et al., 2018; Wu et al., 2020).

In regard to the “quality” of knowledge exchange for the network (Filieri and Willison, 2016), the high relational embeddedness means that members only need to interact with a small number of other members (Gilsing and Duysters, 2008; Chung, 2012), and there is a low demand for knowledge integration in all relevant fields (Dezi et al., 2019), and the types of knowledge exchanged are usually that have compiled and easily transferred (Gilsing and Duysters, 2008; Chung, 2012). Such type of network pattern is more suitable for a stable task environment (Dezi et al., 2019). Usually, the knowledge transfer is a cross-departmental knowledge integration (Aribi and Dupouët, 2015; Ferraris et al., 2018; García-Villaverde et al., 2018), which needs to gather the knowledge of the value chain in each production activity, so that the strength of the network is high (Evangelista and Hau, 2009; Wu et al., 2020), and it is conducive to the transfer of relevant knowledge (Evangelista and Hau, 2009). Hence, the higher the relational embeddedness is, the less chance individual members have with other members to make the production of knowledge (Moran, 2005; Nielsen, 2005; Park and Glaister, 2009; Squire et al., 2010).

The cooperation boundaries are clearly marked by legal contracts among enterprises (Chen et al., 2013), and the development of the relationship among them is also restricted by such legal contracts (Dezi et al., 2019). But these boundaries can be broken by the embedded social relationship (Gilsing and Duysters, 2008; Chung, 2012). Relational embeddedness can overcome difficulties, and help reduce the cost of knowledge acquisition. In this process, existing knowledge is exchanged freely, thus facilitating learning (Ferraris et al., 2018; Dezi et al., 2019; Wu et al., 2020). Hansen (1999) argued that weak ties contribute to quickly seeking knowledge and information that are necessary, but they will become a hindrance factor for knowledge transfer if the knowledge is much more complicated and explicit (Evangelista and Hau, 2009; Aribi and Dupouët, 2015; García-Villaverde et al., 2018). Tiwana (2008) considered that enterprises will be able to absorb the ideas and thoughts easily of their counterparts with shared values among network members. This can facilitate the transmission and integration of tacit knowledge and elimination distrust and uncertainties, and make both sides get immersed in the resolution of alliance issues (Wu et al., 2020). If there is a much closer relationship between the enterprise and its partners, both sides would be more willing to transfer to each other, respectively, tacit or complex knowledge (Moran, 2005; Nielsen, 2005; Park and Glaister, 2009; Squire et al., 2010).

Therefore, firms equipped with good relational embeddedness are competent in making comparison between their current knowledge and new network knowledge (Gilsing and Duysters, 2008; Evangelista and Hau, 2009; Heavey et al., 2015; Flor et al., 2018; such firms can obtain knowledge based on continuously repeated reflections (Alinaghian et al., 2020), and work out solutions together with their customers and suppliers (Dezi et al., 2019), resulting in the possession of tacit knowledge (Andersson et al., 2007; Park and Glaister, 2009; Dezi et al., 2019). As indicated by some scholars, a majority of flowing external knowledge is repeated and easily accessible, and firms are difficult to obtain knowledge sources with competitive advantages; the only approach to providing meaningful knowledge basis for the accumulation of absorptive knowledge is to obtain the irreplaceable know-know and tacit knowledge through the close cooperation and relations with partners. To sum up, relational embeddedness is the basis for firms acquiring, converting, and utilizing external knowledge (Alinaghian et al., 2020). In the process of interorganizational learning, external and internal knowledge can be combined, and potential and realized ACs are intensified (García-Villaverde et al., 2018; Wu et al., 2020). Thus, a hypothesis is developed as follows:

*H5: Relational embeddedness positively affects potential absorptive capacity (PAC)*.

*H6: Relational embeddedness positively affects Realized absorptive capacity (RAC)*.

As stated by Tiwana (2008), firms can learn from each other through shared values from network members (Reagans and McEvily, 2003), which can facilitate the transfer and integration of tacit knowledge, eliminate distrust and uncertainties, propel mutual coordination, and promote the solution of problems (Andersson et al., 2007; Lin et al., 2016). When there is a higher inter-firm cognition and social identity, solid bonds will be created to enhance reciprocal knowledge acquisition and reduce the demand for formal supervision (Evangelista and Hau, 2009). As a result, SMEs can put more effort into knowledge absorption and application (Tsai and Ghoshal, 1998). From a brief literature review, we can see that joint problem solving (Andersson et al., 2007), shared values, and bridging ties can all help SEMs to accumulate knowledge sources required to enhance their exploration and exploitation capabilities (Dezi et al., 2019). Furthermore, Cheung et al. (2010) stated that, if there is a closer relationship among organizations, such as trust, complementarity, compatibility, and idiosyncratic investments, which would trigger knowledge and information sharing between them, entering the process of relationship learning (Dezi et al., 2019). In other words, the knowledge and information acquired from dyadic relationship learning will be accumulated and stored in the exclusive knowledge base, thus enriching and improving the functional, environmental, and interactional knowledge stores in relational memory (Ferraris et al., 2018). Through more relational embeddedness, firms can convert the external knowledge, information, and resources from relational learning into the basis of developing internal capability. Based on the above statements, a hypothesis is developed as follows:

*H7: Relational embeddedness positively affects relational memory*.

When there is a high degree of relational embeddability for the manufacturer, a cross-organizational social connection network is formed through interpersonal contact from different levels and departments among partner organizations (Chen et al., 2013; Ferraris et al., 2018). As the mutual trust and familiarity of network members can facilitate the learning activities of partner organizations, such as information sharing, dissemination, and mutual understanding (Dezi et al., 2019), the close social connection network, is formed by a high degree of relationship embeddedness, can also contribute to improving the maintenance of relational performance (Aribi and Dupouët, 2015; García-Villaverde et al., 2018). In addition, buyers and sellers can also gain performance improvement through effective partnership (Alinaghian et al., 2020), that is, partnership with high efficiency can complete transactions in a more rapid, low-cost, and mutually responsive manner by establishing, developing, and maintaining relationships on behalf of an individual organization (Alinaghian et al., 2020; Wu et al., 2020). Through relational learning, partner organizations share and understand information and disseminate it, making it the organization's knowledge store (Ferraris et al., 2018). Partner organizations have a deeper understanding of each other's needs, and deeper involvement in each other's actions and plans. And it further enables suppliers to develop products and services that better meet customers' needs and reduce each other's overall costs of inventory and quality control, thus creating greater value through the learning process of the partnership (Alinaghian et al., 2020). In addition, relational embeddedness also promotes a faster understanding of each other's problems, richer information exchange, and correct response among partner organizations, which contributes to the development of problem solutions (Andersson et al., 2007) and thus a more satisfactory solution for both partners in the partnership is figured out (Alinaghian et al., 2020; Wu et al., 2020).

*H8: Relational embeddedness positively affects relational performance*.

**Figure 1:**
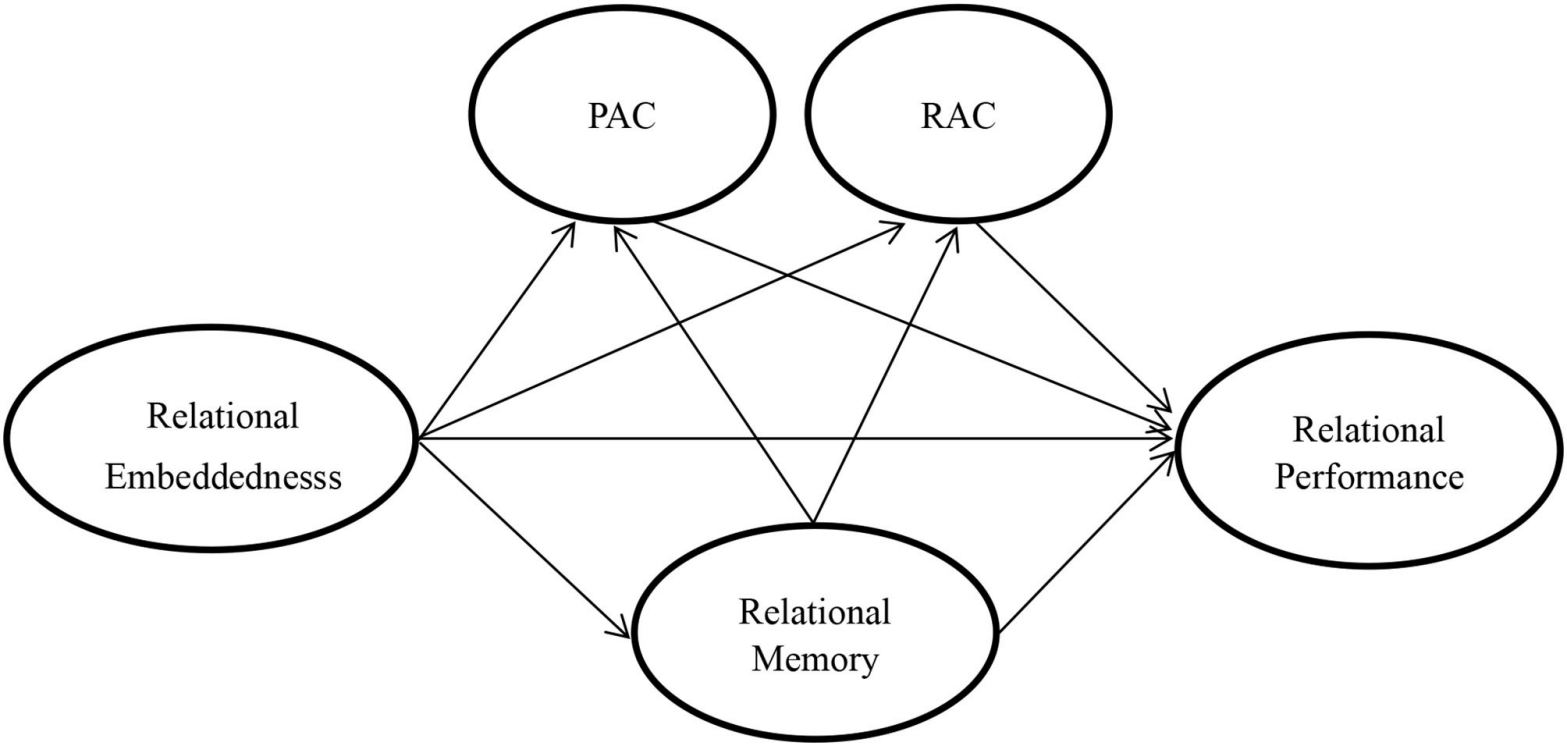
Research framework.

According to the above hypotheses, the research framework is shown in Figure 1.

## Methodology

### Sampling

The target population is composed of internationalized SMEs in mainland China. We selected SMEs from the database using the following criteria: degree of cooperation experiences and the size of organizations (small and medium-sized). Questionnaires were distributed in March 2021 and withdrawn in May 2021, during which the COVID-19 pandemic was wreaking havoc. Although the pandemic has affected business operations and globalization of enterprises, understanding the development of internationalized SMEs in the post-pandemic era will contribute to more appropriate insights and connotations. Large firms were excluded because, in general, they tend to rely on top managers' interpersonal relations to gain access to information and knowledge. Service firms (e.g., consultants) were excluded from our target population. The questionnaire was mailed to 1000 SMEs and 216 copies were recalled in actual. Excluding those who did not complete the questionnaire and those who filled with the same answer, as well as those who did not know the internal and external operations of the organization with <1 year's experience, a total of 4 copies were deleted, and 212 copies were effectively recalled, leading to a response rate of 21.2%.

It takes more than 2 months from the distribution to the withdrawal of questionnaires. We will carry out the non-response bias test in order to identify the possibility of errors in questionnaires withdrawn at different periods. The questionnaires were divided into two groups, recalled and unrecalled, according to the suggestions from Armstrong and Overton (1977), and they tested whether there were significant differences between the two groups in the sample data and the study dimension. Verification results show that there is no significant difference between the main dimensions and the basic information, which indicates that the problem of possible bias from non-response bias is not serious.

Furthermore, the study collects information from the same respondents in form of a single questionnaire, which may lead to the common method bias (CMB). In this study, the single factor verification from Harman is adopted and all the measured items are analyzed by the non-rotating matrix. The analysis results demonstrate that there are nine factors, of which the eigenvalue is >1, and the explanatory variance of factor 1 is 38.42% which could not explain most of the variance. Therefore, it can be concluded from the verification results that there is no common method bias in this study.

### Measures

This study also takes into account the studies of Swaminathan and Moorman (2009). The scale of relational embeddedness was adopted from Dhanaraj et al. (2004), and was measured using a 14-item scale, such as “As we have been doing business for so long, we can understand each other well and quickly” and “As we have been doing business so long, both sides know the weaknesses of the other and do not take advantage of them.”

Consistent with previous work, this study operationalized AC as a higher-order construct of PAC (acquisition and assimilation capacity) and RAC (transformation and application capacity) and adopted scales developed by Camisón and Forés (2010). This measure consists of 19 items: acquisition (4 items), assimilation (5 items), transformation (5 items) and application capacity (4 items), such as “We can capture relevant, continuous and up-to-date information and knowledge on current and potential competitors,” “We can assimilate new technologies and innovations that are useful or have proven potential,” and “We can adapt technologies designed by other to the firm's particular needs.”

Following Johnson et al. (2004), we adopted the following multi-dimensional methods to measure relational memory: functional knowledge store (4 items), environmental knowledge store (4 items), and interactional knowledge store (4 items), such as “Negotiating with suppliers,” “Cost-reduction strategies involving suppliers,” and “Laws and regulations relevant to supplier relationships.”

The items related to relational performance were developed on the basis of the theoretical discussion given in Selnes and Sallis (2003). This study adopted 6 items to measure relational performance, such as “cooperation can generate flexible production based on market fluctuations” and cooperation allows firms to reduce their logistical cost.” All above scales are measured by a 5-point Likert scale ranging from 1 (strongly disagree) to 5 (strongly agree).

## Results

### Assessing the Measurement Model

In order to verify the validity of the measurement model, this study conducted confirmatory factor analysis (CFA) via PLS-SEM to examine the construct validity, including convergent and discriminant validity. Based on validity criteria recommended by Hair et al. (2010), CFA results show that standardized factor loadings were higher than 0.5; average variance extracted (AVE) ranges between 0.50 and 0.82; and composite reliability (CR) ranges between 0.83 and 0.91. As Table 1 shown, all three criteria for convergent validity were met. With regard to discriminant validity, that is, when the square root of the average variance extracted (AVE) is greater than the absolute values of other coefficients relating to the correlation coefficients of this dimension, then the existence of discriminant validity can be supported. The results show that the square root of the average variance extracted is greater than the absolute value of any other coefficient on the same column of the Correlation Coefficient Table, so it can be said that this study has discriminant validity.

**Table 1:** Verification of measurement model.

	**1**	**2**	**3**	**4**	**5**	**6**	**7**	**8**	**9**	**10**	**11**
1. Acquisition											
2. Assimilation	0.363										
3. Transformation	0.315	0.755									
4. Application	0.300	0.712	0.719								
5. Ties	0.279	0.474	0.471	0.455							
6. Trust	0.171	0.490	0.538	0.468	0.595						
7. Shared value	0.214	0.511	0.471	0.418	0.652	0.562					
8. IKS	0.253	0.439	0.371	0.440	0.531	0.380	0.419				
9. FKS	0.277	0.528	0.442	0.515	0.413	0.417	0.384	0.690			
10. EKS	0.267	0.548	0.473	0.549	0.445	0.405	0.473	0.670	0.759		
11. RP	0.155	0.556	0.480	0.450	0.446	0.514	0.469	0.467	0.504	0.501	
Mean	3.560	3.606	3.673	3.622	3.543	3.595	3.552	3.636	3.669	3.545	3.559
SD	0.783	0.673	0.617	0.712	0.753	0.644	0.771	0.688	0.684	0.742	0.708
α	0.832	0.842	0.814	0.807	0.896	0.850	0.887	0.868	0.852	0.860	0.894
AVE	0.685	0.613	0.575	0.635	0.763	0.575	0.749	0.717	0.693	0.705	0.656
CR	0.812	0.888	0.817	0.874	0.928	0.890	0.923	0.910	0.900	0.905	0.919

Henseler et al. (2015) proposed the heterotrait–monotrait (HTMT) ratio of the correlations and suggested 0.90 as a threshold value. As shown in Table 2, the values ranged from 0.445 to 0.888, which indicated that discriminate validity was well-established.

**Table 2:** Discriminant validity: Heterotrsait–monotrait (HTMT).

	**1**	**2**	**3**	**4**	**5**	**6**	**7**	**8**	**9**	**10**	**11**
1. Acquisition											
2. Assimilation	0.857										
3. Transformation	0.790	0.854									
4. Application	0.810	0.869	0.888								
5. Ties	0.610	0.547	0.555	0.539							
6. Trust	0.470	0.578	0.645	0.574	0.685						
7. Shared value	0.450	0.591	0.553	0.494	0.733	0.649					
8. IKS	0.671	0.511	0.445	0.524	0.605	0.442	0.478				
9. FKS	0.710	0.623	0.535	0.624	0.477	0.492	0.442	0.802			
10. EKS	0.628	0.643	0.565	0.658	0.509	0.473	0.593	0.774	0.888		
11. RP	0.539	0.641	0.560	0.535	0.501	0.590	0.527	0.528	0.580	0.571	

### Structural Model

As for the assessment of the structural model, there is a suggestion by Hari et al. (2017) that, based on a subsample of 5,000, the *R*^2^, beta (β), and the corresponding *t*-values are referred to by a bootstrapping algorithm. Not only these basic measures but also the predictive relevance (Q^2^) and the effect sizes (f^2^) should be mentioned by researchers. Furthermore, it is available to make the affirmation the values of the variance inflation factor (VIF) prior to hypotheses testing. And the values of VIF are <5, which are changing from the scope of 1 and 2.032. Hence, no collinearity problems occur in the predictor latent variables (Hari et al., 2017).

**Figure 2:**
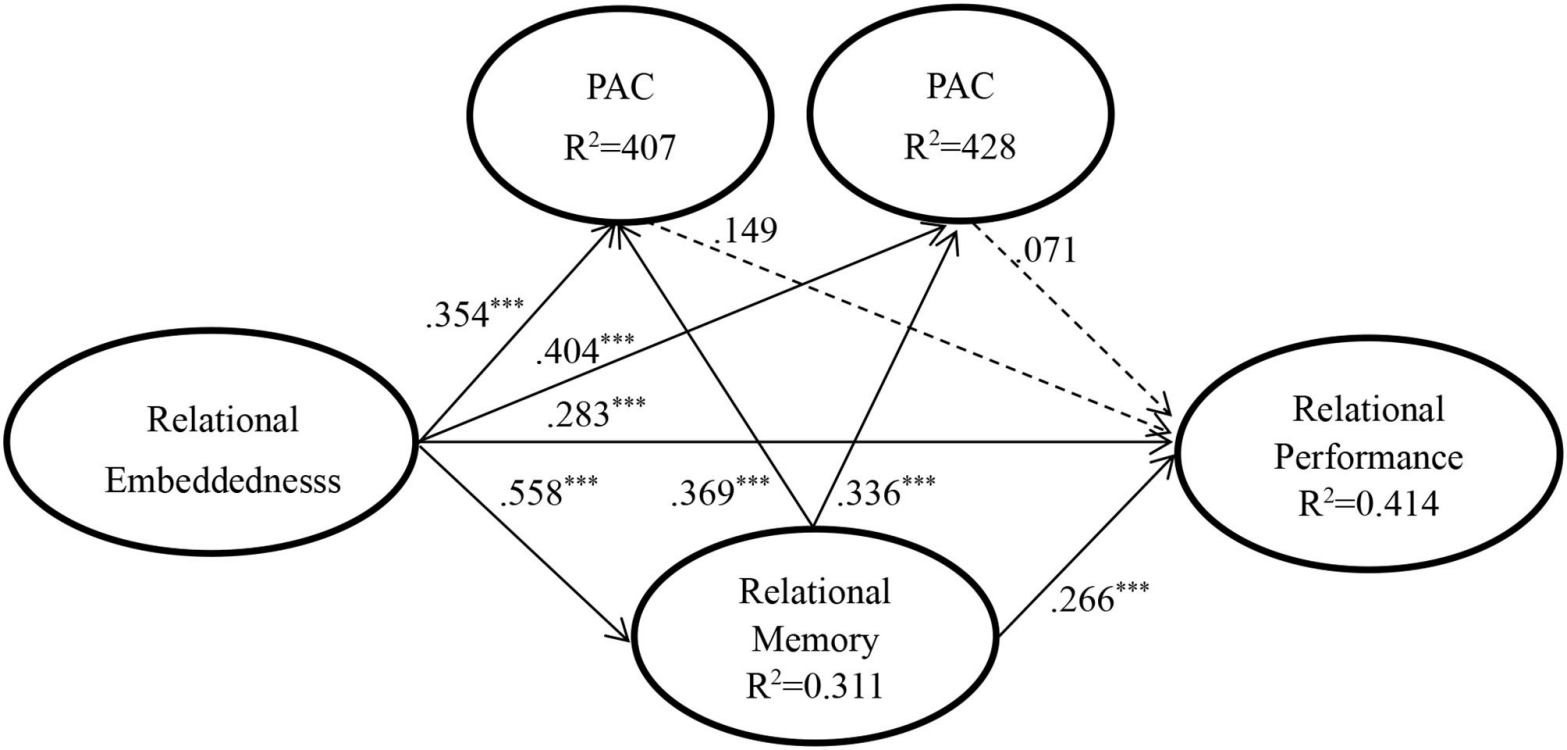
Structural model. ***p < 0.001.

The model explains 41.4% of the variance for relational performance, 31.1% of the variance for relational memory, 40.7% of the variance for PAC and 42.8% of the variance for RAC. Figure 2 shows the results of the hypothesized relationships and standardized coefficients in collected sample. The results showed that PAC (β = 0.149, f^2^ = 0.014, *p* > 0.1) and RAC (β = 0.071, f^2^ = 0.003, *p* > 0.1) were not positively and significantly related to relational performance, not supporting H1 and H2. Relational memory was positively and significantly related to PAC (β = 0.369, f^2^ = 0.158, *p* < 0.001), RAC (β = 0.336, f^2^ = 0.136, *p* < 0.001) and relational performance (β = 0.266, f^2^ = 0.070, *p* < 0.001), supporting H3a, H3b, and H4. Similarly, relational embeddedness was positively and significantly related to PAC (β = 0.354, f^2^ = 0.146, *p* < 0.001), RAC (β = 0.404, f^2^ = 0.196, *p* < 0.001), relational memory (β = 0.558, f^2^ = 0.452, *p* < 0.001) and relational performance (β = 0. 283, f^2^ = 0.077, *p* < 0.001), supporting H5a, H5b, H6, and H7. Therefore, expect H1 and H2, all the hypotheses were supported.

### Analysis of Mediation

Despite there being no mediation hypothesis deduced in this study, under the concept of organizational learning, knowledge, and information absorbed externally need to be transformed so as to be effectively reflected in the performance, so the mediation effect of AC and relational memory has been tested. However, PAC and RAC do not have a significant impact on relational performance, so only the mediation effect of relational memory on relational embeddedness, PAC, RAC, and relational performance has been verified. To calculate the mediation effect, we adopted the formal mediation test proposed by Zhou and Li (2010). As shown in Table 3, first, the indirect effects of relational embeddedness on PAC (β = 0.206, *t* = 4.540), RAC (β = 0.188, *t* = 4.048), and relational performance (β = 0.148, *t* = 3.170) are significant. Second, the direct effects of relational embeddedness on PAC (β = 0.354, *t* = 4.964), RAC (β = 0.404, *t* = 5.281) and relational performance (β = 0.283, *t* = 3.208) are significant. Third, the direct effects and indirect effects operate in the same direction (all are positive). Therefore, partial mediations were confirmed; effects of relational embeddedness on PAC, RAC, and relational performance are partially mediated by relational memory.

**Table 3:** Structural model assessment for direct and indirect effects.

**Effect**	**Std β**	***T*** **-value**
**Direct effects**		
Relational embeddedness → PAC	0.354^***^	4.964
Relational embeddedness → RAC	0.404^***^	5.281
Relational embeddedness → Relational memory	0.558^***^	9.382
Relational embeddedness → Relational performance	0.283^***^	3.208
Relational memory → PAC	0.369^***^	5.034
Relational memory → RAC	0.336^***^	4.205
Relational memory → Relational performance	0.266^***^	3.460
**Indirect effects**		
Relational embeddedness → Relational memory → PAC	0.206^***^	4.540
Relational embeddedness → Relational memory → RAC	0.188^***^	4.048
Relational embeddedness → Relational memory → Relational performance	0.148^**^	3.170

** *p < 0.01;*

*** *p < 0.001*.

## Conclusions

### Discussion

Our results provide some important insights into the role of relational embeddedness from the perspectives of social capital and organizational learning. Although knowledge acquisition and learning through relational embeddedness have become a shot-gun approach for an SME to be accessible to suitable knowledge while improving its knowledge memory, AC, and relational performance that is not easily developed within its confines, the procedure of significant knowledge processing lead to consider the roles of knowledge transformation, internalization and application until the relational performance obtains improvement. Even if relational embeddedness presents high knowledge flow and exchange, there is no guaranteed successful knowledge acquisition for the growth of relational performance, because SMEs may not have sufficient knowledge base to acquire and apply the learned knowledge for the cooperative purpose. This study discusses the relationships among relational embeddedness, relational memory, AC, and relational performance, which has extended recent social capital research (i.e., Camisón and Forés, 2010; Nielsen and Gudergan, 2012; Albort-Morant et al., 2018). Based on organizational learning and social capital perspective, this study proposes a conceptual framework to verify how an SME leverages knowledge/resource and capability to improve their own knowledge base and facilitate it to have more relation-specific knowledge to substantively enhance AC. To be specific, this study gives its inputs from the following aspects.

The data provide strong support for the hypotheses in terms of both statistical and practical significance. First, this study adds support for the positive impacts of relational embeddedness on the relational memory, PAC, and RAC, as stated in previous studies (e.g., Prange and Verdier, 2011; Pinho and Prange, 2016), through the statistical analysis in an Asian context. As a supplement to previous discussions, this study further consolidates the support for social capital and AC from the organizational learning perspective. This study also takes relational embeddedness and relational memory as important antecedents to enhance SMEs' PAC and RAC. This differs from studies of Liu (2012); Limaj and Bernroider (2019), and Rakthin et al. (2016) that consider absorptive capacity as an antecedent, and is contrary to the research framework of Scuotto et al. (2017). Our findings demonstrate that relational embeddedness and relational memory mainly and positively affect relational performance, which is related to the paradigm of the external knowledge acquisition process (Palacios-Marqués et al., 2015). As stated by Cohen and Levinthal (1990), absorptive capacity enables enterprises to acquire, transfer, and assimilate external knowledge within the organization and then generate new ideas (Zahra and George, 2002; Flattena et al., 2011; Kang and Lee, 2016; Limaj and Bernroider, 2019). SMEs with stronger partnerships and a relational knowledge base will be easier to access valuable knowledge sources through more closed connections (Palacios-Marqués et al., 2015), thus providing a solid foundation for absorption of internal knowledge and creating favorable conditions for adapting to changes in the competitive context, especially in AC.

However, research finding shows that PAC and RAC have no statistically significant effects on relational performance, this is inconsistent with and even opposite to the research results of Cepeda-Carrion et al. (2012) and Wu et al. (2020). There are two possible reasons for it. Firstly, knowledge acquired from network or social relations pertains to specific-based knowledge, which can be immediately reflected in the daily operation output of SME, and its output is essentially similar to PAC and RAC, resulting in no significant effect reflected among the three. Furthermore, the formation of PAC and RAC means that an SME should experience a learning process to integrate and combine valuable knowledge with their knowledge stock. For instance, relational performance may also enhance PAC and RAC. Considering the nature of AC, the dynamic perspective of how AC develops is based on a co-evolutionary approach (Zahra et al., 2006; Limaj and Bernroider, 2019). This identifies with the propositions of Wang et al. (2015) that knowledge process-related dynamic capabilities (e.g., PAC and RAC) contain a wider range of knowledge, resource, routine, and relational performance.

Further, our results show that relational memory mediates the positive effects of relational embeddedness on PAC, RAC, and relational performance, whilst there are also direct significant positive effects of relational memory on PAC, RAC, and relational performance. The findings support the argument that SMEs should build dynamic and closed connections with partners and social relations to obtain new knowledge and information about markets and customers to provide certain products and services (Kmieciak, 2019), as a critically important source of relational performance (Albort-Morant et al., 2018). This study presents the concept of relational memory from the perspective of social capital, which is a great contribution to the organizational learning theory. Also, it follows with the claims of Johnson et al. (2004) that if relation-specific knowledge can be stored in an exclusive knowledge store through the learning process within the organization, it would help to improve the integration and application of knowledge and further enhance AC and relational performance. This pattern of findings supports organizational learning theorists' view that the knowledge learning process concerns the acquisition of different information and knowledge, and has a significant impact on the growth of relational performance.

### Managerial Implications

The managerial implications of this study mainly concern how SMEs' relational embeddedness is associated with access to AC. If an SME can acquire ideas on products, improve product functions or create customer-preferred products, it can communicate with international partners through channels such as a partnership. Thus, managers are suggested to participate in the maintenance and reinforcement of social network relationships, so as to enhance relational performance by using multiple relation-specific knowledge.

Although relational embeddedness has facilitated the growth of relational performance to a certain extent, they are also subject to some constraints with the application of information and knowledge. In order to collect intelligence from close social networks and share them in the organization in a more effective manner, SMEs must have sufficient knowledge store to interpret them. This study suggests that managers should establish a series of relational knowledge process flow, which can be separated from general knowledge so as to reduce the processing cost or transaction cost arising from repetitive knowledge.

As the concept of market orientation proposed by Cadogan et al. (2003, 2009), the PAC and RAC are still required to enhance the understanding of the market through the collection and application of international information. The process of interpretation relies on the existing PAC and RAC. Moreover, we suggest that managers should accumulate development requirements of PAC and RAC through formal (e.g., internet, integrated information system, business intelligence, and electronic communication system,) and informal (banquets, inter-departmental personal relationships, and information-sharing social network) knowledge-processing mechanisms.

### Research Limitations and Suggestions for Future Studies

Most of the prior studies focus on antecedents of dynamic capabilities, but few of them have discussed the formation factors of AC. Aside from the relational embeddedness discussed in this study, there are also many other important supports for the improvement of international performance, such as long-term relationships and coordination. Moreover, data was collected for this study through highly subjective cross-section questionnaires, leading to the lack of objective data such as business turnover, amounts of export, return on investment, and supplier amount when considering the impact factors of AC. Therefore, future studies are suggested to collect more diversified objective data from enterprises involved to make the analysis results more persuasive.

The huge industrial difference can significantly affect SMEs. However, only SMEs in a single industry are taken into account in this study, and the impact of the industrial difference is not examined. Therefore, future studies are recommended to include SMEs of different industries to ensure the universality of research findings. Current studies suggest PAC and RAC are dominant AC. Existing studies only discuss micro, small and medium-sized B2B firms. Thus, it is recommended to consider large B2B firms in future studies to examine whether they also have similar organizational learning behaviors.

As demonstrated in prior studies, the issue of resource allocation and trade-off still exists between PAC and RAC, although they are the measuring variables of AC. That's why ambidexterity is valued. We suggest further analyzing the moderation of PAC and RAC and identifying whether the ambidextrous context in SMEs can strengthen the cooperation between PAC and RAC. In addition, the sampling base in this study only covers some specific SMEs, so future studies can consider different types of SMEs for a different analysis to examine the possible effect of heterogeneity. Therefore, we suggest including a variety of control variables to conduct a different analysis and confirm a better cross-validation.

## Data Availability Statement

The raw data supporting the conclusions of this article will be made available by the authors, without undue reservation.

## Ethics Statement

The studies involving human participants were reviewed and approved by Academic Committee of School of Business, Foshan University. The patients/participants provided their written informed consent to participate in this study.

## Author Contributions

All authors contributed to conception and design of the study. All authors contributed to the article and approved the submitted version.

## Conflict of Interest

The authors declare that the research was conducted in the absence of any commercial or financial relationships that could be construed as a potential conflict of interest.

## Publisher's Note

All claims expressed in this article are solely those of the authors and do not necessarily represent those of their affiliated organizations, or those of the publisher, the editors and the reviewers. Any product that may be evaluated in this article, or claim that may be made by its manufacturer, is not guaranteed or endorsed by the publisher.
